# Dual effects of biochar and hyperaccumulator *Solanum nigrum* L. on the remediation of Cd-contaminated soil

**DOI:** 10.7717/peerj.6631

**Published:** 2019-03-25

**Authors:** Kang Li, Baoshan Yang, Hui Wang, Xiaohan Xu, Yongchao Gao, Yidan Zhu

**Affiliations:** 1 School of Water Conservancy and Environment, University of Jinan, Jinan, Shandong, China; 2 Shandong Provincial Engineering Technology Research Center for Ecological Carbon Sink and Capture Utilization, Jinan, Shandong, China; 3 Ecology Institute, Qilu University of Technology (Shandong Academy of Sciences), Jinan, Shandong, China

**Keywords:** Available Cd, Plant growth, *Solanum nigrum*, Biochar amendment

## Abstract

Biochar was widely developed for the soil amendment and remediation of heavy metal contaminated soil. The Cd hyperaccumulator, *Solanum nigrum* L., has been paid much more attention with the wide application of phytoremediation. The effects of biochar on the growth and accumulation capacity of *Solanum nigrum* L. in Cd contaminated soil have not been explored so far. The objectives of this study were to explore the dual effects of biochar addition on available Cd in the soil and hyperaccumulation of Cd in* Solanum nigrum* L. under different Cd contaminated levels. The correlations of soil physicochemical and biochemical properties and Cd absorption of *Solanum nigrum* L. were analyzed after a 60-day pot experiment under three biochar doses (0%, 1% and 5%) and four Cd concentrations (0, 25, 50 and 100 mg kg^−1^). The availability of Cd obtained by DTPA extraction significantly decreased after biochar application (*P* = 0.003, *P* = 0.0001, *P* = 0.0001 under 1% biochar addition for 25, 50, and 100 mg kg^−1^ Cd concentrations, *P* = 0.0001, *P* = 0.0001, *P* = 0.0001 under 5% biochar addition for 25, 50, and 100 mg kg^−1^ Cd concentrations, *n* ≥ 3). The 1% biochar dose significantly increased leaf dry weight (*P* = 0.039, *P* = 0.002 for the Cd concentrations of 50 and 100 mg kg^−1^, *n* ≥ 3) compared with the control in higher Cd concentrations (50 and 100 100 mg kg^−1^). In the presence of biochar, the bioconcentration factor (BCF) increased under the Cd concentrations of 50 and 100 mg kg^−1^. The translocation factors (TF) decreased with the biochar doses under the Cd concentration of 100 mg kg^−1^. The dose of 5% biochar significantly increased the urease activity by 41.18% compared to the 1% biochar addition in the Cd contaminated soil of 50 mg kg^−1^ concentration. The activities of acid phosphatase were inhibited by 1% biochar dose in all the Cd contaminated soils. The dry weight of the root of *Solanum nigrum* L. was significantly negatively correlated with acid phosphatase activity and BCF, respectively, indicating acid phosphatase in the rhizosphere soil of *Solanum nigrum* L. were repressed by Cd toxicity despite of biochar amendment. Biochar had no negative effect on Cd accumulation ability of *Solanum nigrum* L. Two-way ANOVA analysis showed that both biochar and Cd significantly affected the height of *Solanum nigrum* L. and the dry weight of leaf and stem. This study implied that biochar addition does not limit the absorption of hyperaccumulator *Solanum nigrum* L. in the remediation of Cd-contaminated soil. This study implied that the simultaneous application of biochar and hyperccumulator *Solanum nigrum* L. is promising during the remediation of Cd-contaminated soil.

## Introduction

Heavy metals have been considered one of the serious pollution sources in soil because of the excessive accumulation with the development of agriculture and industry. Considering the non - degradability and widespread distribution, the remediation of heavy metal contaminated soil have attracted more attention during soil health management (Sidhu et al., 2017). As one efficient, cost-effective and environment-friendly method, phytoremediation has been focused on extensive interest for the purification of heavy metals contaminated soil and water (Garbisu & Alkorta, 2001). Some plant species, known as hyperaccumulators, have the ability to tolerate toxic metals and accumulate heavy metals without any obvious toxicosis (Buendía-González et al., 2010). *Solanum nigrum* L., a proved Cd hyperaccumulator, is an annual or perennial plant and is widely distributed around the world due to its remarkable ecological adaptability (Khan et al., 2014).

Biochar (BC) is one of the by-products that derived from various feedstocks at high pyrolyzed temperature in limited or absolutely absent oxygen conditions (Lehmann, 2007). The biochar could increase carbon sink, sustain soil nutrients, improve water use efficiency and boost crop yield, thus, it is prevalent in the soil amendment (Wang et al., 2017). Biochar usually has many affinity characteristics such as microporous structures, large specific area with various functional groups, and high ion exchange capacity (Tang et al., 2012; Shaaban et al., 2018; Liu et al., 2018). Biochar addition was advocated as an effective soil remediation techniques in pervasive heavy metal contaminated sites (Kavitha et al., 2018; Wang et al., 2018).

Phytoextraction and biochar immobilization were widely applied to remedy the heavy metal contaminated soil (Rees et al., 2015). The purpose is to immobilize available heavy metals by biochar and remove the rest available fraction by phytoextraction. Therefore, the holistic understanding of the functions of both biochar and hyperaccumulators in the remediation of heavy metal contaminated soil is necessary. Furthermore, the integrated effects of biochar and the hyperaccumulator *Solanum nigrum* L. on soil remediation are very limited.

We made the hypothesis that although biochar reduced the availability of Cd in the soil, it did not significantly affect the enrichment ability of *Solanum nigrum* L. due to the promotion of the growth of *Solanum nigrum* L. In this study, our objectives were: (1) to explore effect of biochar application on the growth of *Solanum nigrum* L.; (2) to investigate the effects of biochar addition on the Cd availability in the soil and bioconcentration factor of *Solanum nigrum* L.; (3) to detect the correlation between bioconcentration factors and soil physicochemical and biochemical properties resulting from biochar addition.

## Materials & Methods

### Soil collecting and biochar preparation

Soil samples used in this experiment were collected in a wheat farmland located in Jinan city, Shandong Province, China (116°57′E, 36°36′N) in March, 2017. The 0–20 cm soil layer was excavated with a steel shovel. One part of the soil was passed through a 2 mm sieve and air dried for soil physicochemical properties. The other was sieved to 4 mm to conduct the pot experiment. The primary basic properties of the soil were measured according to *He et al. (2016)* and are listed in Table S1. The total Cd content was below the upper threshold (0.2 mg kg^−1^) of level 1 of the Chinese environmental quality standard for soils (GB 15618-1995).

The selected soil was artificially contaminated at 25, 50, 100 mg Cd per kg dry soil (supplied with CdCl_2_. 2.5H_2_O), respectively. The polluted soil was preincubated at 25 °C in the dark for eight weeks. The soil was not aging as a historically Cd-contaminated soil. The biochar was produced from corncob at 300 °C under limited air condition according to the method of *Cao et al. (2016)*. The prepared biochar was cooled to room temperature inside the furnace and then passed through a 2 mm sieve for the homogenized sizes. The values of pH and EC were measured in a suspension of biochar at the ratio of 1: 20 (biochar: water = 1: 20, w/v) using pH and EC meter. The specific surface areas and pore volume determined by N_2_-BET measurements. The elemental compositions (C, H and N) and basic properties of the biochar were depicted in Table S1.

### Pot experiment and treatment

The seeds of *Solanum nigrum* L. used in this study were purchased from Germplasm Resources Bank of Kunming Plant Research Institute, Chinese Academy of Sciences. The selected qualified seeds were sterilized with 75% ethanol solution for 30 s then thoroughly flushed with sterile deionized water. After they were dried with sterile filter paper, the seeds were placed in climatic chamber to cultivate in sterilized sand at 12 h light/12 h night and 15/25 °C. *Solanum nigrum* L. seedlings were transferred into pots when they grow two or three leaves (three seedlings in each pot).

A cultivation experiment was carried out in March 2017 in the greenhouse of University of Jinan, Shandong province, China. The average temperature of the greenhouse ranged from 21 °C to 27 °C. The plastic pots (15 cm height ×20 cm diameter) were filled with 1.7 kg of soil (equivalent to weight dry). Biochar was thoroughly mixed with Cd contaminated soil at three proportions of 0%, 1% and 5% (weight/weight) and then the biochar amended soil was incubated for one week before *Solanum nigrum* L. seedling was transplanted into the soil. The soil moisture was kept 50% of maximal water-holding capacity (WHC). After one week of acclimatization, one seedling was maintained in each pot. This experiment was adopted a free combination design with four Cd concentrations and three biochar doses. Four replicates were arranged per treatment. The biochar treatments were referred to as BC-1, and BC-5, respectively. Contaminated soil that was not amended with biochar was served as the control (BC-0). The pot experiments were conducted with a natural day/night regimen in the greenhouse. The soil moisture content was maintained at about 50% of WHC by weighting the pot and adding sterile distilled water.

### Plant growth and Cd content analysis

*Solanum nigrum* L. were collected and divided into roots, stems and leaves 60 days after the transplantation. Roots were first washed with tap water and then were rinsed with EDTA-Na_2_ solution to remove Cd ions adsorbed on the root surface. Finally, the roots were rinsed with distilled water. Plant height was measured and recorded. The fresh biomass of each plant tissue was weighed. The dry weighs were determined according to plant oven-dried method (105 °C for 15 min and 70 °C until stable weights).

Dried plant tissues were finely ground with a quartz mortar. The above treated samples were digested with a mixture of HNO_3_-HClO_4_ and Cd content was determined by atomic absorption spectrometry (AAS). The total heavy metal content in the soil was analyzed using a HNO_3_ - HCl digestion followed by AAS. Briefly, 0.250 g (± 0.005 g) of finely ground soil sample was digested with nitric acid and hydrochloric acid. The digested solution was mixed thoroughly for 10 s using a vortex. Then it stood overnight and was transfer to 50 mL volumetric flask for AAS analysis.

The rhizosphere soil of *Solanum nigrum* L. was collected from the soil attached to the roots after manually shaking them to remove loosely attached soil aggregates. The available Cd in soil samples was extracted with diethylenetriamine pentaacetic acid (DTPA) (0.005 M DTPA, 0.01 M CaCl_2_ and 0.1 M triethanolamine at pH 7.3) according to the methods descried by *Mohamed et al. (2015)*. The concentrations of available Cd were measured by the flame atomic absorption spectrometry (SHIMADIV AA-7000, Japan).

To study the accumulation of *Solanum nigrum* L. in different Cd polluted soil, bioconcentration factor (BCF) and translocation factor (TF) was calculated according to *Sidhu et al. (2017)*.

(1) \begin{eqnarray*}\mathrm{BCF}={\mathrm{C}}_{\mathrm{shoot}}/{\mathrm{C}}_{\mathrm{soil}}\end{eqnarray*}

(2) \begin{eqnarray*}\mathrm{TF}={\mathrm{C}}_{\mathrm{shoot}}/{\mathrm{C}}_{\mathrm{root}}\end{eqnarray*}

Where, C_shoot_, C_root_ and C_soil_ represent Cd concentrations in shoot, root, and soil, respectively.

### Soil physicochemical properties and enzyme activities analysis

Soil EC values were measured in slurry at 2.5: 1 (w/w) ratio. The pH values, TOC, ammonium, and nitrate were analyzed according to He et al. (2016). Sucrase activity was assayed according to Gu, Wang & Kong (2009) with slight modification. In brief, 5 g of rhizosphere soil was mixed with toluene and phosphate (pH 5.5). Then 8% sucrose solution was added. After 24 h shaking at 37 °C for 24 h, the solution was filtered. Finally, 3, 5-dinitrosalicylic acid monohydrate was added for spectroscopic measurement at 508 nm. Urease activities were analyzed using the methods as described by Gu, Wang & Kong (2009). Acid phosphatase activity was measured referred as Chen et al. (2014).

### Data analysis

The data were recorded and primarily analyzed by Microsoft Excel 2010. All the mean values of treatment samples were subject to multiple mean comparisons using the least significant difference (LSD) test at *P* = 0.05 through SPSS of Version 19.0. The interaction of biochar and Cd was analyzed with two-way ANOVA test. All the figures were produced using SigmaPlot 12.0.

## Results

### Changes of soil pH, EC and SOC

As shown in Table 1, *Solanum nigrum* L. as well as the plant biochar could increase pH values in the Cd contaminated soil. The soil pH value of the untreated soil was 6.64, which is faintly acidic, but the soil amendments of 1% biochar dose increased pH values to 7.02–7.37 due to the alkalinity of biochar and the difference was significant between the treatments of biochar and the control without biochar addition under the Cd concentration of 50 mg kg^−1^(*P* = 0.002).

**Table 1:** Changes of soil pH, EC, and SOC after the amendments of biochar and *Solanum nigrum* L. in Cd –contaminated soil.

Treatment^1^	pH	EC (µS/cm )	SOC (g/kg)	NO_3_^−^-N (mg/kg)	NH_4_^+^-N (mg/kg)
Untreated soil	6.64 ± 0.08	31.5 ± 2.14	14.59 ± 0.89	5.49 ± 0.29	6.48 ± 0.06
Cd25 + BC-0	6.97 ± 0.18a	132.40 ± 21.80b	16.13 ± 2.06a	1.86 ± 0.75a	4.77 ± 0.31b
Cd25 + BC-1	7.04 ± 0.03a	127.93 ± 6.61b	20.74 ± 3.70a	1.81 ± 0.53a	9.63 ± 4.18a
Cd25 + BC-5	6.85 ± 0.10b	241.33 ± 3.77a	22.83 ± 2.63a	0.85 ± 0.11a	4.90 ± 1.34b
Cd50 + BC-0	7.09 ± 0.06b	142.30 ± 6.81b	11.7 ± 0.38b	3.86 ± 2.27a	6.94 ± 0.05a
Cd50 + BC-1	7.37 ± 0.00a	151.53 ± 10.79b	19.44 ± 0.94a	1.97 ± 0.59a	5.93 ± 0.16a
Cd50 + BC-5	6.75 ± 0.03a	274.33 ± 33.41a	19.13 ± 0.65a	2.09 ± 0.53a	5.91 ± 0.07a
Cd100 + BC-0	6.79 ± 0.06b	149.47 ± 15.33b	18.78 ± 1.34a	15.80 ± 1.00a	7.09 ± 0.41a
Cd100 + BC-1	7.02 ± 0.03b	178.77 ± 17.36b	19.62 ± 0.20a	8.73 ± 2.58b	6.39 ± 0.48a
Cd100 + BC-5	7.30 ± 0.09a	353.33 ± 62.51a	18.56 ± 0.59a	13.38 ± 1.77a	6.08 ± 0.25a

**Notes.**

Cd25, Cd50, and Cd100 present the Cd concentrations of 25 mg kg^−1^, 50 mg kg^−1^ and 100 mg kg^−1^, respectively; BC-0, BC-1, and BC-5 mean the biochar doses of 0%, 1% and 5% (weight/weight); Values represent the mean ± standard error (*n* = 3), different letters indicate significant differences among different biochar treatments within the same Cd concentration at a significance level of 0.05.

EC significantly increased with 5% biochar for all soil Cd concentrations. There was no significant difference between 0% and 1% biochar (Table 1). Similar to the changes in EC values, SOC increased after the addition of biochar in the same contaminated level except for the 5% biochar dose in the highest Cd concentration of 100 mg kg^−1^. The SOC contents were 28.58% and 41.54% higher than only in the plant system under the Cd concentration of 25 mg kg^−1^, respectively (Table 1).

### The plant growth

Dry weight and plant height per pot under different treatments are shown in Table 2. When the soil was amended with biochar, the biomass of *Solanum nigrum* L. increased or decreased compared with the control without biochar. The 1% biochar dose was found to increase dry weight of the different parts compared with the control in all Cd contaminated soils and the increasing effects on leaves and roots were significant in the soils with higher Cd concentrations (50 mg and 100 mg Cd in per kg dry soil) (*P* = 0.04 and *P* = 0.002 for the leaf of 50 and 100 mg kg^−1^ Cd concentrations; *P* = 0.04 and *P* = 0.02 for the root of 50 and 100 mg kg^−1^ Cd concentrations, *n* ≥ 3) (Table 2). The application of 5% biochar increased the plant dry weight by 11.11%, 60.00%, 47.37% for leaf, stem and root dry weight under the Cd concentration of 50 mg kg^−1^ when compared with the control (without biochar addition), but the differences of leaf and stem were not significant (*P* = 0.087 and *P* = 0.378 for leaf and stem, *n* ≥ 3). The addition of biochar significantly increased the height of *Solanum nigrum* L. compared with the control under the higher Cd concentrations (50 and 100 mg kg^−1^) (*P* = 0.0001 and *P* = 0.0001 under 1% biochar addition for 50 and 100 mg kg^−1^ Cd concentrations, *n* ≥ 3; *P* = 0.0001 and *P* = 0.0001 under 5% biochar addition for 50 and 100 mg kg^−1^ Cd concentrations, *n* ≥ 3) (Table 2). The height of *Solanum nigrum* L. increased with the proportion of biochar at the treatment under low Cd concentration of 25 mg Cd kg^−1^(*P* = 0.003, *n* ≥ 3). However, for the higher Cd concentrations of 50 and 100 mg kg^−1^, the heights were inhibited by 5% biochar addition compared with the treatment of 1% biochar (Table 2). The inhibition rates were 7.90% and 20.70%, respectively. Two-way ANOVA analysis showed that both biochar and Cd significantly affected the height of *Solanum nigrum* L. and the dry weight of leaf and stem (Table 3).

**Table 2:** The dry weight of leaf, stem and root and the mean height of *Solanum nigrum* L. under different treatments of biochar in Cd contaminated soils.

Cd (mg/kg)	Biochar (%)	Leaf (mg/plant)	Stem (mg/plant )	Root (mg/plant)	Height (cm)
0	0	0.97 ± 0.03a	0.51 ± 0.03b	0.54 ± 0.13a	29.49 ± 2.61a
	1	1.13 ± 0.03a	0.96 ± 0.13a	0.54 ± 0.09a	44.42 ± 3.52b
	5	1.05 ± 0.08a	1.16 ± 0.14a	0.65 ± 0.23a	46.17 ± 3.10b
25	0	0.95 ± 0.20a	0.80 ± 0.32a	0.44 ± 0.17a	40.88 ± 3.81a
	1	1.08 ± 0.16a	1.01 ± 0.27a	0.49 ± 0.14a	44.38 ± 1.79a
	5	0.83 ± 0.22a	0.96 ± 0.14a	0.40 ± 0.12a	53.56 ± 2.03b
50	0	0.81 ± 0.20b	0.45 ± 0.12b	0.19 ± 0.10b	37.96 ± 3.60a
	1	1.05 ± 0.11a	1.51 ± 0.89a	0.33 ± 0.12a	53.70 ± 1.61b
	5	0.89 ± 0.05b	0.72 ± 0.03b	0.28 ± 0.04a	49.41 ± 5.25b
100	0	0.18 ± 0.08b	0.09 ± 0.03a	0.05 ± 0.02b	20.08 ± 3.08a
	1	0.55 ± 0.17a	0.21 ± 0.15a	0.21 ± 0.15a	37.38 ± 1.99b
	5	0.30 ± 0.07b	0.09 ± 0.01a	0.09 ± 0.01a	29.64 ± 4.09b

**Notes.**

Values are means (±SE) of four replicate pots in the biochar addition experiment. Different lowercase letters indicate the significant differences among biochar doses at same Cd concentration at *P* <0.05.

**Table 3:** Two-way ANOVA test showing *F*-value of parameters of *Solanum nigrum* (L.) affected by biochar (0, 1 and 5%) and Cd concentration (0, 25, 50 and 100 mg kg^−1^).

Parameter	BC	Cd	BC × Cd
Leaf (DW)	12.06^***^	64.87^***^	1.07ns
Stem (DW)	9.18^**^	19.41^***^	3.20^*^
Root (DW)	2.34ns	29.94^***^	0.55ns
Height	83.41^***^	80.12^***^	6.70^***^
Cd concentration in the shoot	0.08ns	168.55^***^	0.88ns
Cd concentration in the root	0.78ns	40.67^***^	0.28ns
BCF	0.51ns	120.48^***^	0.57ns
TF	3.18ns	26.24ns	1.43ns

**Notes.**

Significant differences are reported as ns, *P* > 0.05.

* *P* < 0.05.

** *P* < 0.01.

*** *P* < 0.001.

### The available Cd in the soil

**Figure 1:**
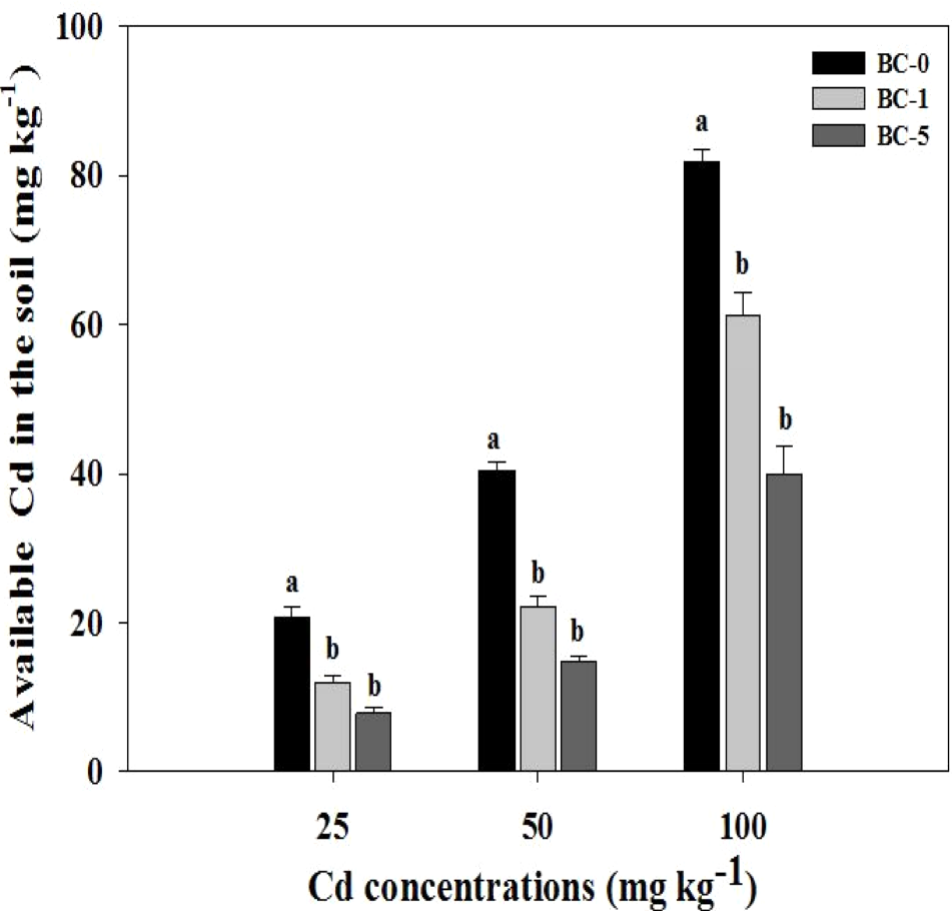
The changes of available Cd after biochar amendment. The effects of biochar addition on available Cd in different Cd contaminated soils. BC-0, BC-1 and BC-5 indicate biochar doses of 0%, 1% and 5%, respectively. Different letters indicate the significant differences among biochar amendments in the same Cd concentration at *P* < 0.05. The error bar is standard deviation.

There was an obvious regular change of available Cd concentrations in rhizosphere soil after the application of biochar (Fig. 1). The soil used in the experiment was not ageing soil, which may increase the virtual available Cd before the biochar was added in the soil. The contents of available Cd decrease with increasing proportion of biochar regardless of Cd levels in the soil. The addition of 5% biochar dose reduced available Cd contents more than that of 1% biochar dose (62.40% and 63.60% under 5% and 42.90% and 45.1% under 1% biochar dose in Cd contaminated soils of 25 and 50 mg kg^−1^, respectively). Under the Cd concentration of 100 mg kg^−1^, the treatments under 1% and 5% biochar reduced bioavailable Cd by 25.00% and 51.10%.

### Phytoextraction and plant Cd contents

The effects of the different biochar treatments on the concentrations of Cd in the shoots and roots of *Solanum nigrum* L. plants are illustrated in Table 4. The Cd concentrations in shoots (*P* = 0.548, *P* = 0.234, *P* = 0.988 under 1% biochar addition under 25, 50, and 100 mg kg^−1^ Cd concentrations, *n* ≥ 3; *P* = 0.483, *P* = 0.096, *P* = 0.661 under 5% biochar addition under 25, 50, and 100 mg kg^−1^ Cd concentrations, *n* ≥ 3) and roots (*P* = 0.491, *P* = 0.853, *P* = 0.256 under 1% biochar addition for 25, 50, and 100 mg kg^−1^ Cd concentrations, *n* ≥ 3; *P* = 0.665, *P* = 0.981, *P* = 0.242 under 5% biochar addition for 25, 50, and 100 mg kg^−1^ Cd concentrations, *n* ≥ 3) were not significantly affected by biochar addition (Table 4). Compared with control, the addition of biochar reduced the Cd contents in the shoot under the Cd concentration of 25 mg kg^−1^. However, Cd contents of shoot showed contrary patterns under the Cd concentrations of 50 mg kg^−1^ in the soil. Cd concentrations of the root showed the similar pattern to biochar doses under the Cd concentration of 100 mg kg^−1^ in the soil. The Cd contents in the roots reached a maximum of 600.10 mg kg^−1^ dry weight. The Cd concentrations in the roots of *Solanum nigrum* L. linearly increased with the elevated Cd contents in the soil. We found the similar change pattern that the Cd concentrations in the shoot increased first and decreased with increasing biochar dose under the treatment of Cd contamination of 100 mg kg^−1^. BCF increased with biochar dose in Cd concentration of 50 mg kg^−1^ (Table 4). But the differences of BCF were not significant among the biochar treatments in all Cd contaminated soil (*P* = 0.597, *P* = 0.203, *P* = 0.766 for 1% biochar addition under 25, 50, and 100 mg kg^−1^ Cd concentrations, *n* ≥ 3; *P* = 0.853, *P* = 0.151, *P* = 0.955 for 5% biochar addition with 25, 50, and 100 mg kg^−1^ Cd concentrations, *n* ≥ 3). TF reduced after 1% biochar addition and it increased after 5% biochar application when compared with 1% biochar dose in the Cd contaminated soil of 25 mg kg^−1^ and 50 mg kg^−1^. However, TF reduced with increasing biochar doses under the Cd concentration of 100 mg kg^−1^. Biochar had no significant effect on the Cd concentration of the shoots and roots and BCF. However, Cd level in the soil had a significant effect on the Cd concentrations of the shoots and roots and BCF (Table 2).

**Table 4:** Cd contents, bioconcentration factor (BCF) and translocation factor (TF) of hyperaccumulator (*Solanum nigrum* L.) under different biochar treatments in the soils with different Cd levels.

Cd levels (mg/kg)	Biochar (%)	Cd content (mg/kg)		BCF	TF		
		Shoot	Root		
25	0	254.93 ± 26.05	132.25 ± 5.63	10.23 ± 1.43		1.88 ± 0.37^*^	
	1	223.50 ± 31.63 (*P* = 0.45)	206.10 ± 3.20 (*P* = 0.49)	9.64 ± 2.48 (*P* = 0.59)		1.08 ± 0.20^*^ (*P* = 0.02)	
	5	219.72 ± 5.58 (*P* = 0.48)	178.51 ± 10.75 (*P* = 0.66)	10.43 ± 0.98 (*P* = 0.85)		1.23 ± 0.07 (*P* = 0.64)	
50	0	267.92 ± 22.51	324.88 ± 80.79	5.67 ± 0.56		0.94 ± 0.37	
	1	313.01 ± 1.45 (*P* = 0.23)	344.65 ± 15.59 (*P* = 0.85)	7.11 ± 0.20 (*P* = 0.20)		0.91 ± 0.05 (*P* = 0.91)	
	5	332.22 ± 17.27 (*P* = 0.09)	322.28 ± 77.43 (*P* = 0.98)	7.31 ± 0.63 (*P* = 0.15)		1.14 ± 0.35 (*P* = 0.55)	
100	0	430.50 ± 53.14	473.15 ± 182.11	1.27 ± 0.69		1.27 ± 0.69^*^	
	1	431.05 ± 20.49 (*P* = 0.98)	596.42 ± 55.78 (*P* = 0.25)	4.56 ± 0.38 (*P* = 0.76)		0.73 ± 0.13 (*P* = 0.12)	
	5	414.17 ± 16.49 (*P* = 0.66)	600.10 ± 20.00 (*P* = 0.24)	4.29 ± 0.25 (*P* = 0.95)		0.69 ± 0.04^*^ (*P* = 0.04)	

**Notes.**

Values are means (±SE) in four pots under same treatment.

* indicate significant differences between biochar doses at same Cd concentration at *P* < 0.05.

**Figure 2:**
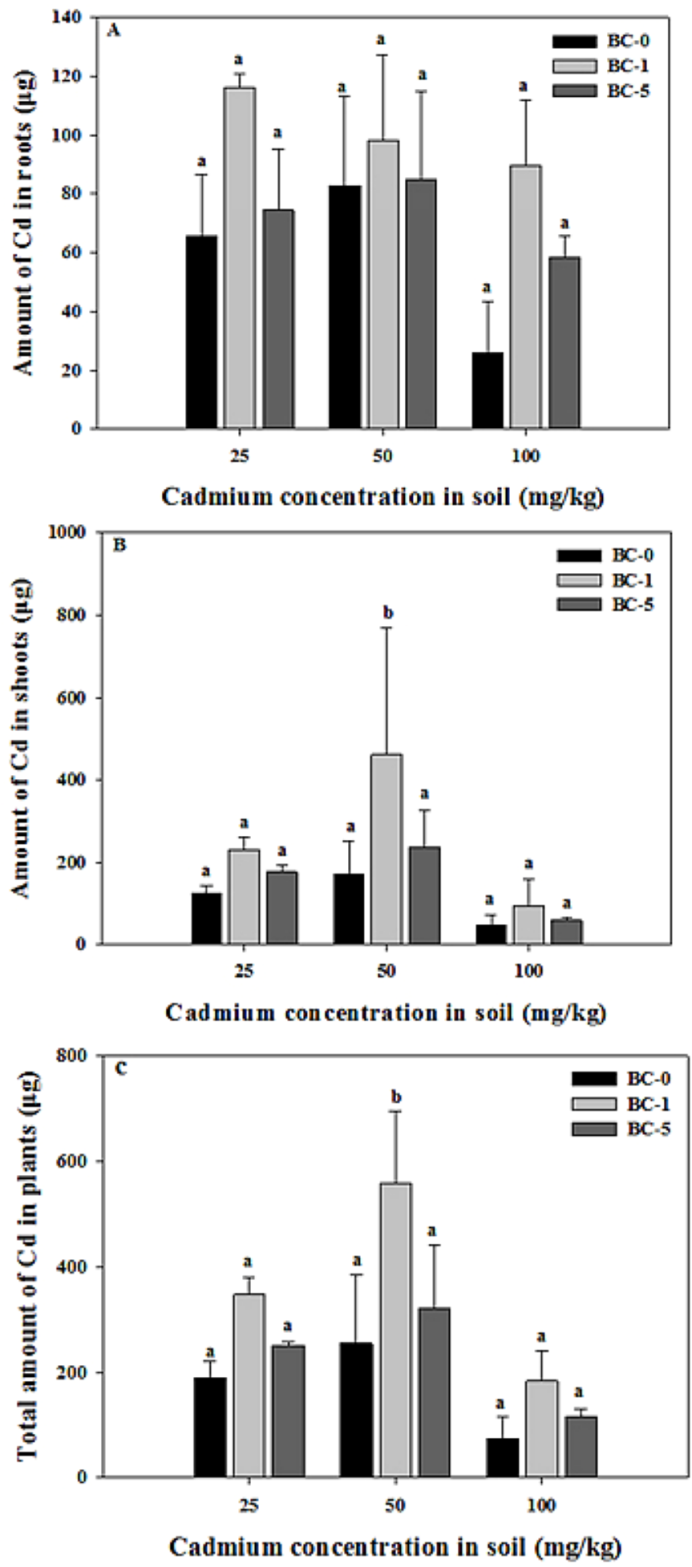
The amount of Cd uptake by roots, shoots and whole plants after biochar addition. The effect of biochar dose on the amount of Cd uptake by roots, shoots and whole plants in different Cd contaminated soil. BC-0, BC-1 and BC-5 indicate biochar doses of 0%, 1% and 5%. Different letters present the significant differences at *P* < 0.05 in the same Cd concentration. The value is mean ± SD (*n* ≥ 3).

As shown in Fig. 2, biochar treatment did not have a significant effect on the amount of Cd in the plants. The total Cd amounts increased first and decreased at the highest Cd concentration of 100 mg kg^−1^ rather than increasing with an increase of external Cd concentration. The Cd amount regardless of being in the roots or shoots was the highest under the 1% biochar amendment in all the contaminated soil. The greatest Cd amount were found in the shoot and whole plant at medium-high Cd concentrations (50 mg kg^−1^) after biochar addition, and the highest Cd levels, 461.15 µg in shoot and 559.20 µg in whole plant, were attained under the treatment of 1% biochar (Fig. 2). The largest Cd content in the root, 115.98 µg, was observed at the Cd concentration of 25 mg kg^−1^ and 1% biochar addition. The shoot is the main part of *Solanum nigrum* L. used to accumulate Cd under the lower Cd concentrations of both 25 mg kg^−1^ and 50 mg kg^−1^.

### Soil enzymatic activity

**Figure 3:**
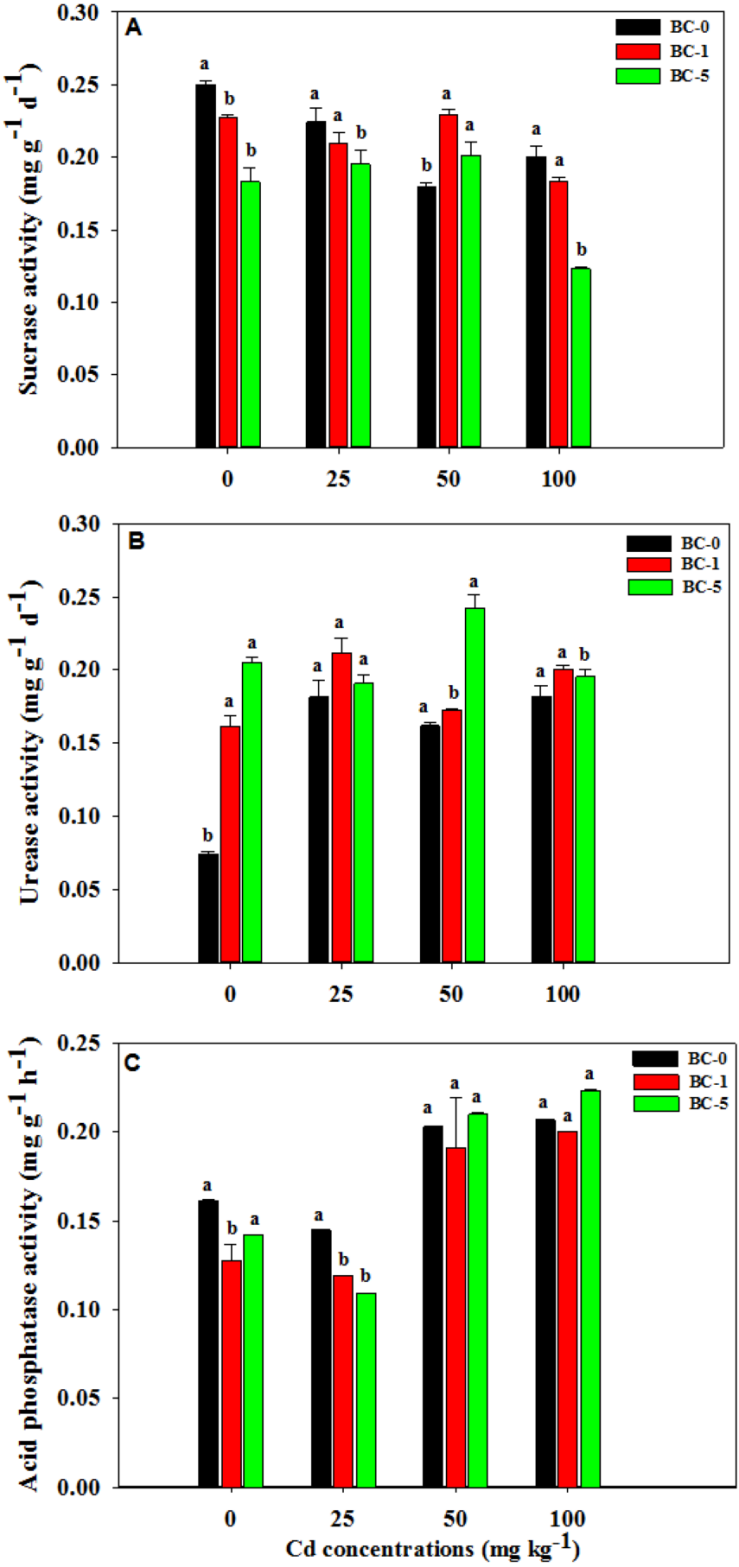
Enzymatic activities after biochar addition. The effects of biochar dose on enzymatic activities in different Cd contaminated soils (mean ± SD, *n* ≥ 3). (A) Sucrase; (B) Urease, (C) Acid phosphatase activity. BC-0, BC-1 and BC-5 indicate the treatments of 0%, 1% and 5% biochar dose, respectively. Bars represent SD of four replicates. Different lower cases indicate significant differences among biochar treatments in the same Cd concentration at *P* < 0.05.

The application of 5% dose biochar significantly inhibited the sucrase activities under the Cd concentration of 0, 25 and 100 mg kg^−1^ compared to the treatment without biochar (*P* = 0.0001, *P* = 0.004 and *P* = 0.0001 under the Cd concentrations of 0, 25 and 100 mg kg^−1^, *n* ≥ (3) (Fig. 3). A positive effect was found under the Cd concentration of 50 mg kg^−1^ in contaminated soil. The stimulation rates were 27.3% and 12.0% for the amendments of 1% and 5% biochar (Fig. 3A). Sucrase activity was the lowest in the application of 5% biochar under the highest Cd concentration of 100 mg kg^−1^. All proportions of biochar had a stimulation effect on urease (Fig. 3B). The dose of 5% biochar significantly increased the urease activity by 41.18% compared to the biochar of 1% under the Cd concentration of 50 mg kg^−1^ (*P* = 0.0001, *n* ≥ 3). The pattern was contrary in the soil under the highest Cd concentration of 100 mg kg^−1^. The dose of 5% biochar stimulated soil acid phosphatase activities while the 1% dose inhibited the activities under higher Cd concentrations (50 mg kg^−1^ and 100 mg kg^−1^) (Fig. 3C). The activities of acid phosphatase significantly decreased by 18.75% and 17.93% after the application of 1% biochar in the uncontaminated soil (*P* = 0.004, *n* ≥ 3) and the Cd concentration of 25 mg kg^−1^ compared with control without biochar amendment (*P* = 0.003, *n* ≥ 3) (Fig. 3C).

### The correlativity among hyperaccumulation, plant biomass and soil enzyme

The BCF were positively correlated with leaf dry weight, stem dry weight, and root dry weight while negatively correlated to the activity of acid phosphatase. The activity of sucrase had positive correlation with stem dry weight while acid phosphatase activities were negatively correlated with all the parameters of plant growth (Table 5).

**Table 5:** Pearson correlation coefficients between bioconcentration factor (BCF), translocation factors (TF) of *Solanum nigrum* (L.), soil enzyme activities and plant dry weight.

Parameters	BCF	TF	Sucrase	Urease	Acid phosphatase	Leaf weight	Stem weight	Root weight
BCF	1							
TF	0.639	1						
Sucrase	0.579	0.602	1					
Urease	0.135	−0.061	−0.049	1				
Acid phosphatase	−0.896^**^	−0.499	−0.0464	−0.009	1			
Leaf dry weight	0774^**^	0.284	0.648	0.103	−0.576	1		
Stem dry weight	0.717^**^	0.223	0.703^**^	−0.026	−0.550	0.874^**^	1	
Root dry weight	0.938^**^	0.464	0.621	0.182	−0.845^**^	0.889^**^	0.789^**^	1

**Notes.**

** means the correlation is significant at the 0.05 level (2-tailed).

## Discussion

Biochar has the multiple roles in the soil amendment including increasing plant biomass and reducing heavy metal toxicity to the plant (Wang et al., 2016). Biochar not only improves soil physicochemical properties but also diminishes the heavy metal availability to the plant (Rees et al., 2015). Generally, the release of exchangeable Ca^2+^, K^+^, Mg ^2+^, Mn^2+^ and Na^+^ from biochar to the soil may provide the nutrient elements for roots or shoots of plants, which stimulate plant growth (Abbas et al., 2017). Furthermore, biochar may reduce the availability of heavy metal by adsorbing to its surface (Mohamed et al., 2015). This decreased the toxicity to the plant and facilitated the production of plant biomass (Rees et al., 2015). The results of our study suggested the available Cd decreased after the addition of biochar, regardless of the Cd concentrations in the soil (25, 50 and 100 mg kg^−1^) (Fig. 1). However, the proportion of bichar had no significant effects on Cd content in shoot and roots as compared to the control. The possible reason is that biochar increases soil pH values (Table 1), which could immobilize heavy metal by precipitation (Rodríguez-Vila et al., 2016). At the same time, the rhizosphere soil may be changed due to the growth of *Solanum nigrum* L., which resulted in the no significant reduction in Cd intake of *Solanum nigrum* L. The findings were consistent with the study of Ahmad et al. (2016), who reported that exchangeable Pb has significantly negative correlation with pH/CEC. Furthermore, the available Cd has negative correlation with the doses of biochar (Fig. 1). These conclusions were also supported by Houben, Evrard & Sonnet (2013a, 2013b), who indicated that available Cd was obviously reduced compared to the control after the application of biochar, and Cd availability to the roots was decreased because of an increasing biochar dose. The previous study also found extractable Cd was notably retained when biochar was added into the soil at the rates of 10, 20 and 40 t ha^−1^ (Bian et al., 2014). Similar results was obtained by *Fellet, Marmiroli & Marchiol (2014)*, who indicated DTPA-Cd was reduced from 5.2 mg kg^−1^ in the control to 1.75–0.92 mg kg^−1^ and 2.42–1.51 mg kg^−1^ after the application of 3% orchard-residues biochar and 5% manure-fir tree biochar, respectively. Possible reasons include the adsorption and surface precipitation of biochar reduced the extractable Cd in the soil.

The changes of Cd concentrations in the shoots and roots were not constant with the increasing biochar doses under different Cd concentration in the soils (Table 4). However, the accumulation of Cd in the root increased with the proportion of biochar amended under the high Cd concentration of 100 mg kg^−1^ in the soil (Table 2). This result indicated that the Cd uptake by *Solanum nigrum* L. was not consistent with the changes of available Cd in the soil when amended with biochar. Possible reasons were that the increasing pH values may result in an enhanced negative charge around the root (Houben, Evrard & Sonnet, 2013b), which may improve the adsorption of Cd on the roots and accelerate the uptake of Cd in *Solanum nigrum* L. On the other hand, biochar stimulated the plant growth, especially the dose of 1% biochar in the present study (Table 2). This finding is similar to that reported by Huang et al. (2018), who showed that plant biomass depended on biochar type and dose, indicating positive effects at low rate of biochar addition (<20%, v/v), but negative effects for higher rates. Our results were also consistent with previous research which were proved by using rice straw biochar, chicken manure and rape straw biochar (Abbas et al., 2017; Zhao et al., 2016).

The capacity of plant uptake of heavy metal was varied among plants, depending on the plant species and heavy metal type (Hou et al., 2017; Leite et al., 2017). Rees et al. (2015) found that the application of biochar will increase the surface area of the root, implying a higher uptake area of root to obtain available metal in the soil. This may promote the uptake of heavy metals in plants, although the availability of heavy metal is generally reduced (Namgay et al., 2010). The results obtained in the present study showed that the BCF of *Solanum nigrum* L. decreased in the dose of 1% biochar and increased with the application of 5% biochar in the soil under the Cd concentration of 25 mg kg^−1^ (Table 2). The trade-off between the decrease of Cd^2+^ availability in the soil and the promotion of Cd^2+^ intake by *Solanum nigrum* L. need to be revealed after biochar addition.

Soil enzymes are important drivers during organic matter decomposition and plays essential roles in nutrient cycling (Tian et al., 2016). Sucrase plays an important role in carbon conversion (Tian et al., 2016). In general, the higher the soil fertility, the higher the sucrase activity. It can not only characterize soil biological activity, but also can be used as an indicator to evaluate soil fertility levels. A large number of studies have shown that acid phosphatase plays a very important role in regulating plant phosphorus nutrition (Zheng et al., 2016; Gascó et al., 2016). Determination of the activity of acid phosphatase can reflect the effect of biochar application on the P supply to *Solanum nigrum* L. Their activities were apt to be affected by the changes of soil habitats and thus the changes of enzyme activity are considered an important indicator of soil quality and often used to evaluate the soil health under human disturbance (Zheng et al., 2016; Gascó et al., 2016). It was reported that the addition of biochar was positively correlated with the activity of urease in the soil (Wang et al., 2015). The inferred reason is that the amendment of biochar enhances soil fertility and water holding capacity (Mierzwa-Hersztek, Gondek & Baran, 2016). The other studies indicated that biochar stimulated soil enzyme activities owing to altering the functions of soil microbial community in the contaminated soil (Wu et al., 2016). The effects of biochar on soil enzyme activity were generally correlated with biochar doses. And the effects of low dose biochar on the activities of soil enzyme were not significantly different compared to the control (Mierzwa-Hersztek, Gondek & Baran, 2016). The application of high biochar doses likewise had an adverse effect on enzyme activity. This may be due to the high absorption capacity of biochar to the enzyme substrate, thus inhibiting enzymatic reaction (Oleszczuk et al., 2014; Tian et al., 2016). In the current study, the sucrase activities were inhibited along with the doses of biochar application except for the contaminated soil with Cd concentration of 50 mg kg^−1^ (Fig. 3A). It was inconsistent with previous results showing that biochar addition can have positive effects on enzyme activities in soils contaminated with heavy metals (Lu et al., 2015). At the same time, biochar can be applied to immobilize heavy metals (Ahmad et al., 2014); (Beesley & Marmiroli, 2011) and promote plant growth (Wang et al., 2018; Liu et al., 2018). When biochar accelerates the growth of *Solanum nigrum* L, the plant releases large amount of root exudation, which reduces metal toxicity in the soil contaminated by Cd of 50 mg kg^−1^. Compared with the treatment of BC-0, similar results were found in acid phosphatase activity in low Cd contaminated soil (25 mg kg^−1^) in this study (Fig. 3C). However, the higher dose of biochar stimulated acid phosphatase activity in soils with higher Cd concentrations (50 and 100 mg kg^−1^) (Fig. 3C). In the current study, negative significant Pearson’s correlation coefficients (r) were obtained between acid phosphatase activity and root dry weight (*r* =  − 0.845, *P* = 0.001) or BCF (*r* =  − 0.896, *P* = 0.001) (Table 5), indicating acid phosphatase activities in the rhizosphere soil of *Solanum nigrum* L. were repressed by Cd toxicity despite biochar amendment. The urease activities indicated lower values in the control soil without biochar amendment than in the soils amended with biochar, especially with the higher dosage of 5% biochar (Fig. 3B). In this study, there are no regular changes of soil enzymes activities after the biochar amendment in Cd contaminated soil. However, the changes of enzyme activities were associated with biochar dose and Cd concentrations in the soil. The 5% biochar addition stimulated the activity of acid phosphatase when compared with 0% biochar under the Cd concentrations of 50 and 100 mg kg ^−1^. Thus, the enzyme activities, which were used to indicate the effects of biochar amendment on the improvement of soil biochemical properties, varied with the Cd contaminated level.

## Conclusions

Biochar enhance the Cd contents of shoot and root at the Cd concentration of 50 mg kg^−1^ as well as the BCF. BCF were significantly negatively correlated with acid phosphatase activities. The application of biochar promoted the growth of *Solanum nigrum* L. in uncontaminated soil. The 1% biochar addition has a positive effect on the growth of *Solanum nigrum* L. in the Cd contaminated soil. Biochar has no negative effect on Cd accumulation ability of *Solanum nigrum* L. This implies that the simultaneous application of biochar and hyperaccumulator *Solanum nigrum* L. is adoptable in the remediation of Cd contaminated soil.

##  Supplemental Information

10.7717/peerj.6631/supp-1 Table S1 The properties of biochar and soil

10.7717/peerj.6631/supp-2 Data S1 Raw Data

## Additional Information and Declarations

### Data Availability

The following information was supplied regarding data availability: The raw data is available in Tables 1–5, Figs. 1–3 and in the Supplemental Files.
