# Supplementary material for: Dual effects of biochar and hyperaccumulator Solanum nigrum L. on the remediation of Cd-contaminated soil
Source: PeerJ. 2019 Mar 25;7:e6631. doi: 10.7717/peerj.6631 (PMC6438157; doi:10.7717/peerj.6631)
Supplement: Table S1 [file peerj-07-6631-s001.doc]

Table S1 The properties of biochar and soil.

| Property | Soil | Biochar |
| --- | --- | --- |
| Soil organic carbon (g/kg) | 14.59 | - |
| Available phosphorus (mg/kg) | 48.56 | - |
| NO_3_^–^-N(mg/kg) | 5.49 | - |
| NH_4_^+^-N(mg/kg) | 6.48 | - |
| pH | 6.64 | 9.8 |
| C (%) | - | 67.39 |
| H (%) | - | 3.54 |
| N (%) | - | 1.69 |
| EC (dS/m) | - | 3.15 |
| Specific surface areas (m^2^ /g) | - | 31.567 |
| Pore volume (m^3^ /g) | - | 0.0194 |
